# Parent-of-Origin Effects of the *APOB* Gene on Adiposity in Young Adults

**DOI:** 10.1371/journal.pgen.1005573

**Published:** 2015-10-09

**Authors:** Hagit Hochner, Catherine Allard, Einat Granot-Hershkovitz, Jinbo Chen, Colleen M. Sitlani, Sandra Sazdovska, Thomas Lumley, Barbara McKnight, Kenneth Rice, Daniel A. Enquobahrie, James B. Meigs, Pui Kwok, Marie-France Hivert, Ingrid B. Borecki, Felicia Gomez, Ting Wang, Cornelia van Duijn, Najaf Amin, Jerome I. Rotter, John Stamatoyannopoulos, Vardiella Meiner, Orly Manor, Josée Dupuis, Yechiel Friedlander, David S. Siscovick

**Affiliations:** 1 Braun School of Public Health, Hebrew University-Hadassah Medical Center, Jerusalem, Israel; 2 Département de Mathématiques, Université de Sherbrooke and Centre de Recherche du Centre Hospitalier Universitaire de Sherbrooke, Sherbrooke, Quebec, Canada; 3 Department of Biostatistics and Epidemiology, University of Pennsylvania School of Medicine, Philadelphia, Pennsylvania, United States of America; 4 Department of Medicine, University of Washington, Seattle, Washington, United States of America; 5 Cardiovascular Health Research Unit, University of Washington, Seattle, Washington, United States of America; 6 Department of Statistics, University of Auckland, Auckland, New Zealand; 7 Department of Biostatistics, University of Washington, Seattle, Washington, United States of America; 8 Department of Epidemiology, University of Washington, Seattle, Washington, United States of America; 9 Harvard Medical School and General Medicine Division, Massachusetts General Hospital, Boston, Massachusetts, United States of America; 10 Institute of Human Genetics, University of California, San Francisco, California, United States of America; 11 Cardiovascular Research Institute, University of California, San Francisco, California, United States of America; 12 Department of Dermatology, University of California, San Francisco, California, United States of America; 13 Department of Population Medicine, Harvard Pilgrim Health Care Institute, Boston, Massachusetts, United States of America; 14 Department of Genetics, Washington University School of Medicine, St. Louis, Missouri, United States of America; 15 Genetic Epidemiology Unit, Department of Epidemiology, Erasmus Medical Center, University Medical Center, Rotterdam, the Netherlands; 16 Institute for Translational Genomics and Population Sciences and Department of Pediatrics, Los Angeles BioMedical Research Institute at Harbor-UCLA Medical Center, Torrance, California, United States of America; 17 Department of Genome Sciences, University of Washington, Seattle, Washington, United States of America; 18 Department of Genetics and Metabolism, Hebrew University-Hadassah Medical Center, Jerusalem, Israel; 19 Department of Biostatistics, Boston University School of Public Health, Boston, Massachusetts, United States of America; 20 New York Academy of Medicine, New York, New York, United States of America; Dartmouth College, UNITED STATES

## Abstract

Loci identified in genome-wide association studies (GWAS) of cardio-metabolic traits account for a small proportion of the traits' heritability. To date, most association studies have not considered parent-of-origin effects (POEs). Here we report investigation of POEs on adiposity and glycemic traits in young adults. The Jerusalem Perinatal Family Follow-Up Study (JPS), comprising 1250 young adults and their mothers was used for discovery. Focusing on 18 genes identified by previous GWAS as associated with cardio-metabolic traits, we used linear regression to examine the associations of maternally- and paternally-derived offspring minor alleles with body mass index (BMI), waist circumference (WC), fasting glucose and insulin. We replicated and meta-analyzed JPS findings in individuals of European ancestry aged ≤50 belonging to pedigrees from the Framingham Heart Study, Family Heart Study and Erasmus Rucphen Family study (total N≅4800). We considered p<2.7x10^-4^ statistically significant to account for multiple testing. We identified a common coding variant in the 4^th^ exon of *APOB* (rs1367117) with a significant maternally-derived effect on BMI (β = 0.8; 95%CI:0.4,1.1; p = 3.1x10^-5^) and WC (β = 2.7; 95%CI:1.7,3.7; p = 2.1x10^-7^). The corresponding paternally-derived effects were non-significant (p>0.6). Suggestive maternally-derived associations of rs1367117 were observed with fasting glucose (β = 0.9; 95%CI:0.3,1.5; p = 4.0x10^-3^) and insulin (ln-transformed, β = 0.06; 95%CI:0.03,0.1; p = 7.4x10^-4^). Bioinformatic annotation for rs1367117 revealed a variety of regulatory functions in this region in liver and adipose tissues and a 50% methylation pattern in liver only, consistent with allelic-specific methylation, which may indicate tissue-specific POE. Our findings demonstrate a maternal-specific association between a common *APOB* variant and adiposity, an association that was not previously detected in GWAS. These results provide evidence for the role of regulatory mechanisms, POEs specifically, in adiposity. In addition this study highlights the benefit of utilizing family studies for deciphering the genetic architecture of complex traits.

## Author Summary

To date, genetic variants identified in large-scale genetic studies using recent technical and methodological advances explain only a small proportion of the genetic basis of obesity, diabetes and other cardiovascular risk factors. These studies were typically conducted in samples of unrelated individuals. Here we utilize a family-based approach to identify genetic variants associated with obesity-related traits. Specifically, we examined the separate contribution of maternally- vs. paternally-inherited common genetic variants to these traits. By examining 1250 young adults and their mothers from Jerusalem, we show that a specific genetic variant, rs1367117, located in the *APOB* gene on chromosome 2 is related to body mass index and waist circumference when inherited from mother and not from father. This maternal effect is not restricted to Jerusalemites, but is also seen in a large sample of individuals of European descent from independent family studies worldwide. Our findings provide support of the role of complex genetic mechanisms in obesity, and highlight the benefit of utilizing family studies for uncovering genetic pathways underlying common risk factors and diseases.

## Introduction

Genome-wide association studies (GWAS) have identified multiple loci associated with cardio-metabolic risk (CMR) phenotypes and traits, such as adiposity and glycemic traits (e.g [1–4]). GWAS are conducted, very largely, in unrelated individuals. In general, the magnitudes of these genetic associations are small to modest, and the loci account for only a small proportion of the heritability of these traits [5–8]. To explain the “missing” heritability, various research strategies for follow-up studies have been proposed, including focusing on gene-environment interactions, on rare variants with moderate effects and on parent-of-origin effects (POEs) [7, 9].

POEs are non-Mendelian transmittable genetic effects on phenotypes, where the phenotype in offspring depends on whether transmission originated from the mother or father. In mammals, POEs can be caused by genomic imprinting, maternal genetic effects on the intrauterine environment, or maternally inherited mitochondrial genes [10]. The most obvious mechanism underlying POEs is genomic imprinting; imprinted genes show parental-specific monoallelic or partial expression, dictated by the parental origin of the chromosome [11, 12].

In the past decade, linkage studies have demonstrated POEs on both binary and continuous CMR outcomes [13–17]. Yet, despite evidence for POEs from linkage studies, most association studies have overlooked their potential influence on CMR traits, and the extent and magnitude of POEs on CMR traits remain largely unknown. One important exception, from Iceland, demonstrated that inclusion of the parental source of alleles significantly strengthened previously-reported associations with type 2 diabetes [18]. Two subsequent studies in Icelanders detected POEs on age at menarche and thyroid-stimulating hormone levels, traits that are associated with CMR [19, 20].

To examine POEs on adiposity and glycemic traits in young adults, we leveraged existing genotype data and CMR trait measurements on mother-offspring pairs from a longitudinal study nested within a historical birth cohort, together with GWAS and bioinformatics databases. We replicated our findings using extended pedigrees from three family studies. Our hypothesis was that, in genes where variants are known to be associated with CMR traits, knowledge of the parental source of genetic variants would reveal POEs on body mass index (BMI), waist circumference (WC), and fasting glucose and insulin levels.

## Results

Distribution of JPS offspring adiposity and glycemic traits at mean age 32 and characteristics of the 18 genes and 182 tag SNPs selected for this analysis in JPS are presented in the S1–S4 Tables. All SNPs were common (minor allele frequency (MAF)>0.06) and not far from Hardy–Weinberg equilibrium (p>10^−5^). Associations between offspring genotypes, with and without considering parental source of alleles, for the 182 SNPs and the four traits BMI, WC, fasting glucose and fasting insulin are presented in S5 Table. Nominally significant POEs for adiposity and glycemic traits were observed in JPS. Consequently, 30 of the 182 SNPs initially tested for POE in the JPS were moved forward for replication in the 3 other family studies (S6 Table). In the joint analysis of results from JPS, FHS, FamHS and ERF, significant POEs were observed for a variant in the *APOB* gene (Table 1).

**Table 1:** Parent-of-origin effects of *APOB* SNP rs1367117^a^ on adiposity and glycemic traits.

		Maternally-derived effect			Paternally-derived effect			
Phenotype	No. of probands	Beta	SE^b^	p-value	Beta	SE^b^	p-value	P-value for maternal vs. paternal effects
**BMI, kg/m** ^**2**^	** **	** **	** **	** **	** **	** **	** **	** **
JPS (discovery)	1235	1.437	0.527	0.0065	0.005	0.511	0.9927	0.0856
FHS	2228	0.791	0.250	0.0015	-0.219	0.242	0.3650	0.0033
FamHS	773	1.319	0.436	0.0025	-0.197	0.428	0.6455	0.0112
ERF	622	-0.288	0.414	0.4863	0.340	0.413	0.4107	0.2791
Combined (Z based)	4858			7.8x10-6			0.6185	0.0005
Combined (inverse variance based)	4858	0.752	0.180	3.1x10-5	-0.087	0.176	0.6204	
**Waist circumference, cm**								
JPS (discovery)	1235	3.822	1.270	0.0027	-0.646	1.249	0.6049	0.0260
FHS	2225	2.231	0.649	0.0006	-0.244	0.632	0.6995	0.0056
FamHS	773	3.911	1.187	0.0010	0.279	1.161	0.8101	0.0254
ERF	631	-3.037	3.110	0.3289	-0.834	3.084	0.7868	0.6111
Combined (Z based)	4864			1.6x10-6			0.6007	0.0010
Combined (inverse variance based)	4864	2.660	0.512	2.1x10-7	-0.227	0.501	0.6502	
**Fasting glucose, mg/dL**								
JPS (discovery)	1068	0.342	1.160	0.7682	-0.757	0.984	0.4417	0.5156
FHS	2153	1.315	0.382	0.0006	-0.523	0.380	0.1694	0.0005
FamHS	761	0.036	0.653	0.9560	-1.070	0.681	0.1163	0.2425
ERF	608	0.128	1.062	0.9041	0.279	1.050	0.7905	0.9208
Combined (Z based)	4590			0.0102	-0.522		0.0636	0.0127
Combined (inverse variance based)	4590	0.875	0.304	0.0040	-0.586	0.301	0.0520	
**Fasting insulin, mU/mL (natural log transformed)**								
JPS (discovery)	1102	0.104	0.059	0.0790	-0.038	0.066	0.5585	0.1640
FHS	1960	0.051	0.024	0.0356	0.004	0.027	0.8823	0.1597
FamHS	761	0.129	0.046	0.0049	-0.006	0.049	0.9016	0.0321
ERF	510	0.012	0.044	0.7856	0.017	0.044	0.6999	0.9376
Combined (Z based)	4333			0.0004	0.004		0.6347	0.0745
Combined (inverse variance based)	4333	0.062	0.018	0.0007	0.001	0.020	0.9566	

JPS, Jerusalem Perinatal Study; FHS, Framingham Heart Study; FamHS, Family Heart Study; ERF, Erasmus Rucphen Family

^a^ Minor allele frequency (MAF) of SNP rs1367117 across studies: JPS 0.2, FHS 0.31, FamHS 0.34 (imputed data, R^2^ = 0.925), ERF 0.31.

^b^ Standard errors (SEs) were calculated directly in JPS; in the other studies SEs were estimated by converting p-values into a z-statistic and setting: SE = beta/z.

### Adiposity and glycemic traits

**Fig 1:**
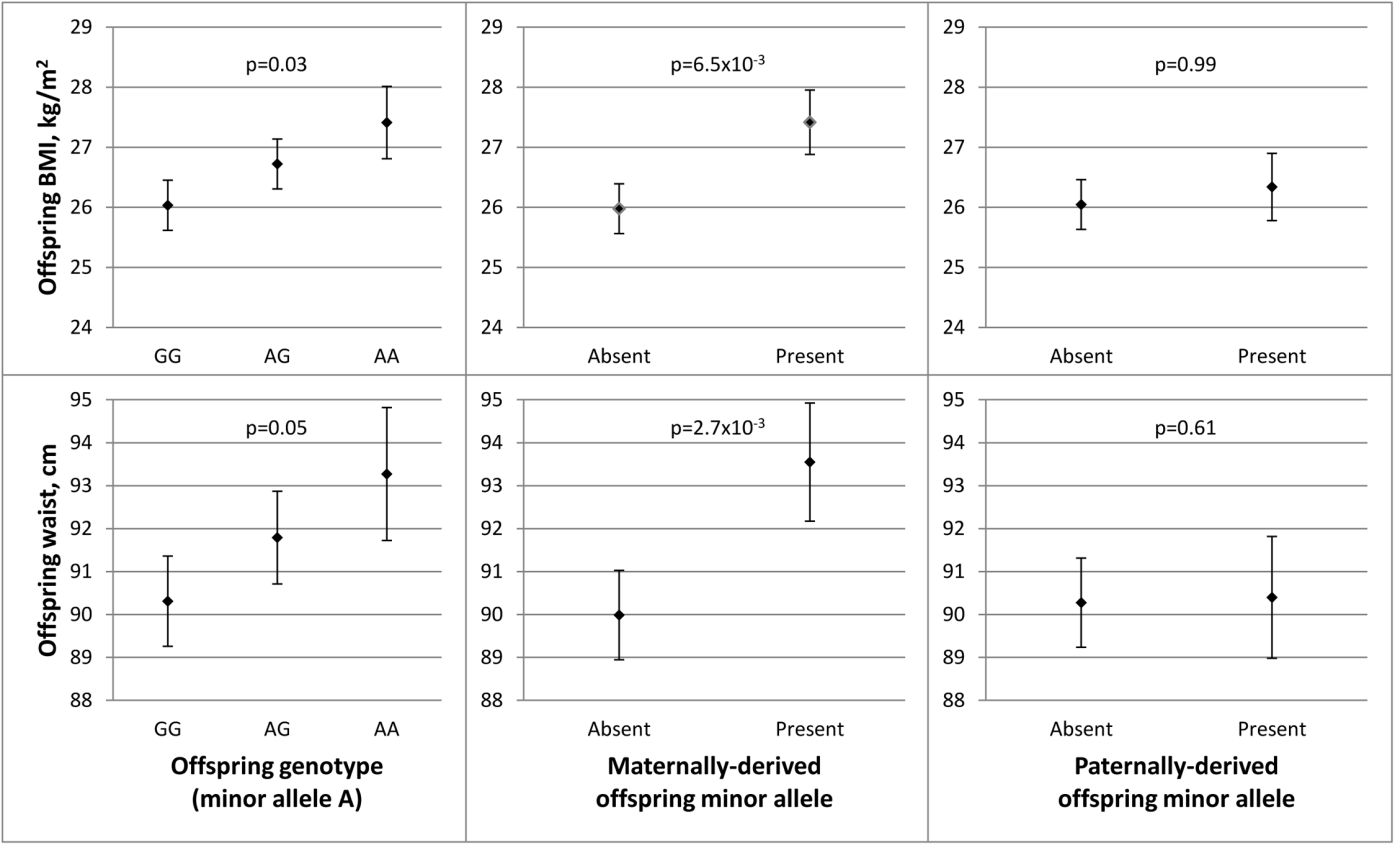
Parent-of-origin effects of *APOB* SNP rs1367117 on adiposity traits in JPS. This figure illustrates the associations between *APOB* SNP rs1367117 and offspring BMI (top panel) and waist circumference (WC) (bottom panel). Adjusted means and standard errors (represented by error bars) for BMI and WC by genotype were determined using estimates from linear regression models adjusted for ethnicity and gender. Comparing offspring genotype effect (left panel), maternally-derived effect (middle panel) and paternally-derived effect (right panel) reveals a strengthened and more significant maternal-specific association with both traits.

In the discovery stage conducted in JPS, SNP rs1367117, a coding SNP located within the *APOB* gene, demonstrated significant POEs on adiposity traits (Table 1 and S5 Table). Specifically, significant associations were demonstrated when the minor allele was inherited from the mother for offspring BMI (beta = 1.44, p-value = 6.5x10^-3^) and WC (beta = 3.82, p-value = 2.7x10^-3^). The corresponding paternally-derived effects were non-significant (p>0.6). As expected in the setting of POEs, in JPS associations seen with the maternally-derived minor allele were approximately twice as strong as those that did not consider the parental source of the allele (effects sizes were 0.7 (p-value = .03) and 1.5 (p-value = .05) for BMI and WC respectively) (Fig 1 and S5 Table). In fact, the differences in simple heritability estimates in JPS derived from mother-offspring correlations [21, 22] based on models with either adjustment for POE or additive genetic effects of the *APOB* variant suggested that POE of the *APOB* SNP explains roughly 0.30% and 0.58% of BMI and WC heritability, respectively. No changes in heritability estimates for BMI or WC were observed for the additive genetic effect of this variant.

We also conducted sensitivity analyses in which we excluded heterozygous mother-offspring pairs (191 of 1235 pairs) instead of using probability-based estimated dosage in these pairs. These analyses resulted in similar or larger estimates for *APOB* rs1367117 POE on BMI (maternal beta = 1.46; p = .048), WC (maternal beta = 5.26; p = .003) and fasting insulin (natural log transformed, maternal beta = .212; p = .004) compared to those using estimated dosage (Table 1) suggesting that our probability-based estimated dosage approach is likely conservative. Additionally, we used simulations to assess the likelihood of false positive findings. Permutation-based p-values for the maternal effect on BMI (p-value = .0079) and on WC (p-value = .0048) were very close to the observed maternal p-values (p-value = .0065 and .0027 for BMI and WC, respectively). Detailed information on simulations in JPS (and in FHS) can be found in S1 Text (permutation testing).

Testing for POEs for *APOB* SNP rs1367117 in the replication and meta-analysis stage, also showed a significant maternally-derived effect in FHS and FamHS separately, but not in ERF, and in all four studies combined on both BMI (combined beta = 0.75, p-value = 3.1x10^-5^) and WC (combined beta = 2.66, p-value = 2.1x10^-7^) (Table 1). The effect sizes for the maternal-specific associations in JPS and FamHS were essentially identical, whereas the corresponding effects in FHS were somewhat smaller. The associations of the paternally-derived allele with those traits were not significant (F-test p-values for difference between maternally- and paternally-derived effects were 5.0x10^-4^ and 1.0x10^-3^ for BMI and WC respectively).

Maternally-derived associations of this *APOB* SNP with borderline significance were also seen with fasting glucose and insulin levels in the combined results (combined p-value = 4.0x10^-3^ and 7.4x10^-4^ for glucose and insulin respectively), whereas no associations were observed with the paternally-derived allele (Table 1).

### Lipids, lipoproteins and blood pressure traits

Because *APOB* is a major component of lipoprotein particles and an important contributor to the atherosclerotic process [23, 24], we examined whether there is also evidence for POEs of *APOB* rs1367117 on other CMR traits, including LDL-C, HDL-C, total cholesterol (TC), triglycerides (TG) and systolic and diastolic BP (S7 Table). A maternally-derived association, and not paternally-derived, was observed for SBP in the combined analysis (combined beta = 1.62, p-value = 1.6x10^-4^; F-test p-value for difference = 3.1x10^-3^). Significant maternal and paternal associations, of similar magnitudes, were shown with both LDL-C (maternal beta = 4.20, p-value = 2.8x10^-6^; paternal beta = 3.54, p-value = 5.0x10^-5^) and TC (maternal beta = 4.67, p-value = 3.6x10^-6^; paternal beta = 3.67, p-value = 1.7x10^-4^) (F-test p-values for difference between maternal and paternal effects were non-significant), reflecting offspring genotype effect, irrespective of the parental source (additive genetic effect is discussed below).

### Mediating role of BMI

To further explore the observed parental-specific associations of *APOB* with CMR traits, we examined the influence of offspring BMI on these associations. Further adjustment for BMI resulted in attenuation of the observed maternally-derived effects of *APOB* rs1367117 on waist, SBP, glucose and insulin to the null (Table 2). In contrast, adjustment for BMI had minimal effect on the maternal and paternal associations with both LDL-C and TC (S7 Table).

**Table 2:** Maternally-derived effects of *APOB* SNP rs1367117 on cardio-metabolic traits with and without adjustment for BMI^a^.

		Maternally-derived effect					
Phenotype	No. of Probands	Beta	SE^b^	p-value	BMI adj. p-value	P-value for maternal vs. paternal effects	BMI adj. p-value for maternal vs. paternal effects
**Waist circumference, cm**							
JPS (discovery)	1235	3.822	1.270	0.0027	0.2181	0.0260	0.1523
FHS	2225	2.231	0.649	0.0006	0.1701	0.0056	0.8296
FamHS	773	3.911	1.187	0.0010	0.2082	0.0254	0.8083
ERF	631	-3.037	3.110	0.3289	0.4057	0.6111	0.8153
Combined (Z based)	4864			1.6x10-6	0.0792	0.0010	0.7606
Combined (inverse variance based)	4864	2.660	0.512	2.1x10-7	0.0413		
**Fasting glucose, mg/dL**							
JPS (discovery)	1068	0.342	1.160	0.7682	0.9782	0.5156	0.6741
FHS	2153	1.315	0.382	0.0006	0.0120	0.0005	0.0104
FamHS	761	0.036	0.653	0.9560	0.2988	0.2425	0.8327
ERF	608	0.128	1.062	0.9041	0.8855	0.9208	0.9137
Combined (Z based)	4590			0.0102	0.1813	0.0127	0.2335
Combined (inverse variance based)	4590	0.875	0.304	0.0040	0.0526		
**Fasting insulin, mU/mL (natural log transformed)**							
JPS (discovery)	1102	0.104	0.059	0.0790	0.9918	0.1640	0.7732
FHS	1960	0.051	0.024	0.0356	0.5942	0.1597	0.9164
FamHS	761	0.129	0.046	0.0049	0.2335	0.0321	0.4763
ERF	510	0.012	0.044	0.7856	0.7337	0.9376	0.7110
Combined (Z based)	4333			0.0004	0.3318	0.0745	0.9432
Combined (inverse variance based)	4333	0.062	0.018	0.0007	0.3008		
**Low-density lipoprotein cholesterol, mmol/L**							
JPS (discovery)	1107	6.960	3.729	0.0622	0.1982	0.6177	0.9892
FHS	2205	4.703	1.462	0.0013	0.0050	0.1635	0.5073
FamHS	739	4.624	2.671	0.0834	0.2932	0.3363	0.1129
ERF	586	3.333	1.332	0.0123	0.0124	0.8577	0.7409
Combined (Z based)	4637			2.5x10-6	0.0001	0.5290	0.6090
Combined (inverse variance based)	4637	4.204	0.897	2.8x10-6	4.8x10-5		
**High-density lipoprotein cholesterol, mmol/L**							
JPS (discovery)	1117	-3.016	1.418	0.0337	0.4094	0.0666	0.3049
FHS	2226	-1.048	0.670	0.1179	0.6345	0.0716	0.2717
FamHS	752	-0.073	0.937	0.9379	0.5300	1.0000	0.4899
ERF	589	0.441	0.539	0.4135	0.6493	0.7997	0.8635
Combined (Z based)	4684			0.0634	0.7521	0.1940	0.5691
Combined (inverse variance based)	4684	-0.329	0.370	0.3746	0.9444		
**Total cholesterol, mmol/L**							
JPS (discovery)	1117	6.387	4.178	0.1266	0.2890	0.6563	0.9775
FHS	2230	4.918	1.660	0.0030	0.0112	0.3137	0.8073
FamHS	752	5.873	3.006	0.0507	0.2187	0.8244	0.3870
ERF	589	3.956	1.484	0.0077	0.0108	0.9111	0.8779
Combined (Z based)	4688			6.3x10-6	0.0003	0.8803	0.9552
Combined (inverse variance based)	4688	4.667	1.008	3.6x10-6	0.0001		
**Triglycerides, mmol/L (natural log transformed)**							
JPS (discovery)	1117	0.097	0.061	0.1143	0.6418	0.0709	0.3342
FHS	2242	0.068	0.026	0.0083	0.2193	0.1099	0.6533
FamHS	752	0.017	0.048	0.7214	0.5222	0.4679	0.9343
ERF	590	0.019	0.041	0.6415	0.5632	0.4400	0.3836
Combined (Z based)	4701			0.0037	0.3085	0.1163	0.7473
Combined (inverse variance based)	4701	0.052	0.019	0.0055	0.2846		
**Systolic blood pressure, mmHg**							
JPS (discovery)	1220	1.120	1.145	0.3284	0.9856	0.8311	0.4120
FHS	2234	1.927	0.585	0.0010	0.0172	0.0584	0.2941
FamHS	733	1.376	0.905	0.1282	0.6705	0.0017	0.0262
ERF	593	1.194	1.376	0.3856	0.3959	0.1124	0.1960
Combined (Z based)	4780			0.0003	0.0381	0.0031	0.0640
Combined (inverse variance based)	4780	1.618	0.429	0.0002	0.0276		
**Diastolic blood pressure, mmHg**							
JPS (discovery)	1220	1.236	0.910	0.1743	0.6806	0.8455	0.4691
FHS	2232	0.370	0.439	0.3990	0.8759	0.3699	0.9585
FamHS	733	1.089	0.792	0.1692	0.5975	0.0319	0.1663
ERF	593	1.495	0.908	0.0997	0.1263	0.0830	0.1943
Combined (Z based)	4778			0.0173	0.3932	0.0768	0.3896
Combined (inverse variance based)	4778	0.756	0.330	0.0217	0.4082		

JPS, Jerusalem Perinatal Study; FHS, Framingham Heart Study; FamHS, Family Heart Study; ERF, Erasmus Rucphen Family

^a^ Presented betas and standard errors (SEs) are based on models without adjustment for BMI. P-values are presented for models with and without further adjustment for BMI. Beta and standard errors for BMI-adjusted models as well as for paternally-derived effects are provided in S7 Table.

^b^ Standard errors (SEs) were calculated directly in JPS; in the other studies SEs were estimated by converting p-values into a z-statistic and setting: SE = beta/z.

### Additive genetic effect on cardio-metabolic traits

We have additionally examined the associations of offspring *APOB* rs1367117 with cardio-metabolic traits, overlooking the parental source of the minor allele, in the four studies separately and combined (Table 3). The combined data pointed to a substantial additive genotype effect on lipid traits, mainly on LDL-C and TC, which is in agreement with the aforementioned POE results showing both maternal and paternal effects on LDL-C and TC (S7 Table). On the other hand, genotype effects on adiposity traits (BMI and WC) were relatively minor both in size and significance, emphasizing the maternal-specific nature of the *APOB* rs1367117-adiposity association.

**Table 3:** Genotype effect of APOB SNP rs1367117 on cardio-metabolic traits.

		Genotype effect^a^		
Phenotype	No. of Probands^b^	Beta	SE	p-value
**BMI, kg/m** ^**2**^	** **	** **	** **	** **
JPS (discovery)	1246	0.689	0.310	0.0263
FHS	3666	-0.029	0.138	0.8329
FamHS	2228	0.329	0.173	0.0573
ERF	1968	0.306	0.168	0.0682
Combined (inverse variance based)	9108	0.209	0.087	0.0166
**Waist circumference, cm**				
JPS (discovery)	1246	1.481	0.766	0.0534
FHS	3659	-0.003	0.361	0.9925
FamHS	2227	0.837	0.470	0.0748
ERF	1965	0.670	0.488	0.1702
Combined (inverse variance based)	9097	0.503	0.235	0.0324
**Fasting glucose, mg/dL**				
JPS (discovery)	1077	-0.279	0.657	0.6715
FHS	3542	-0.127	0.205	0.5363
FamHS	2047	0.547	0.286	0.0560
ERF	1830	-0.131	0.428	0.7588
Combined (inverse variance based)	8496	0.053	0.151	0.7280
**Fasting insulin, mU/mL (natural log transformed)**				
JPS (discovery)	1112	0.030	0.036	0.4016
FHS	3229	0.008	0.013	0.5504
FamHS	2045	0.050	0.019	0.0076
ERF	1486	0.009	0.019	0.6154
Combined (inverse variance based)	7872	0.019	0.009	0.0341
**Low-density lipoprotein cholesterol, mmol/L**				
JPS (discovery)	1117	5.771	2.315	0.0128
FHS	3734	2.997	0.770	0.0001
FamHS	1925	0.696	1.079	0.5192
ERF	1802	6.501	1.401	3.50x10^-6^
Combined (inverse variance based)	8578	3.098	0.556	2.45x10^-8^
**High-density lipoprotein cholesterol, mmol/L**				
JPS (discovery)	1127	-0.671	0.946	0.4785
FHS	3771	-0.516	0.358	0.1501
FamHS	1967	-0.482	0.433	0.2660
ERF	1810	-0.963	0.545	0.0774
Combined (inverse variance based)	8675	-0.601	0.238	0.0117
**Total cholesterol, mmol/L**				
JPS (discovery)	1127	5.258	2.609	0.0441
FHS	3778	2.632	0.867	0.0024
FamHS	1967	0.664	1.197	0.5791
ERF	1810	5.855	1.551	1.61x10^-4^
Combined (inverse variance based)	8682	2.768	0.621	8.43x10^-6^
**Triglycerides, mmol/L (natural log transformed)**				
JPS (discovery)	1127	0.013	0.036	0.7119
FHS	3800	0.012	0.014	0.3829
FamHS	2183	0.035	0.018	0.0537
ERF	1810	0.024	0.020	0.2175
Combined (inverse variance based)	8920	0.021	0.009	0.0249
**Systolic blood pressure, mmHg**				
JPS (discovery)	1231	1.253	0.759	0.0990
FHS	3793	0.251	0.314	0.4241
FamHS	1812	-0.055	0.445	0.9019
ERF	1751	0.022	0.653	0.9734
Combined (inverse variance based)	8587	0.233	0.228	0.3060
**Diastolic blood pressure, mmHg**				
JPS (discovery)	1231	1.428	0.580	0.0139
FHS	3791	-0.048	0.232	0.8347
FamHS	1812	-0.178	0.335	0.5954
ERF	1751	0.561	0.372	0.1314
Combined (inverse variance based)	8585	0.154	0.163	0.3436

JPS, Jerusalem Perinatal Study; FHS, Framingham Heart Study; FamHS, Family Heart Study; ERF, Erasmus Rucphen Family

^a^ Based on an additive genetic model.

^b^ Numbers of probands included in the genotype effect analysis are larger compared to the POE analysis due to exclusion of individuals who were non-informative for parental transmission.

### Fine-mapping the APOB parent-of-origin effect on BMI

**Fig 2:**
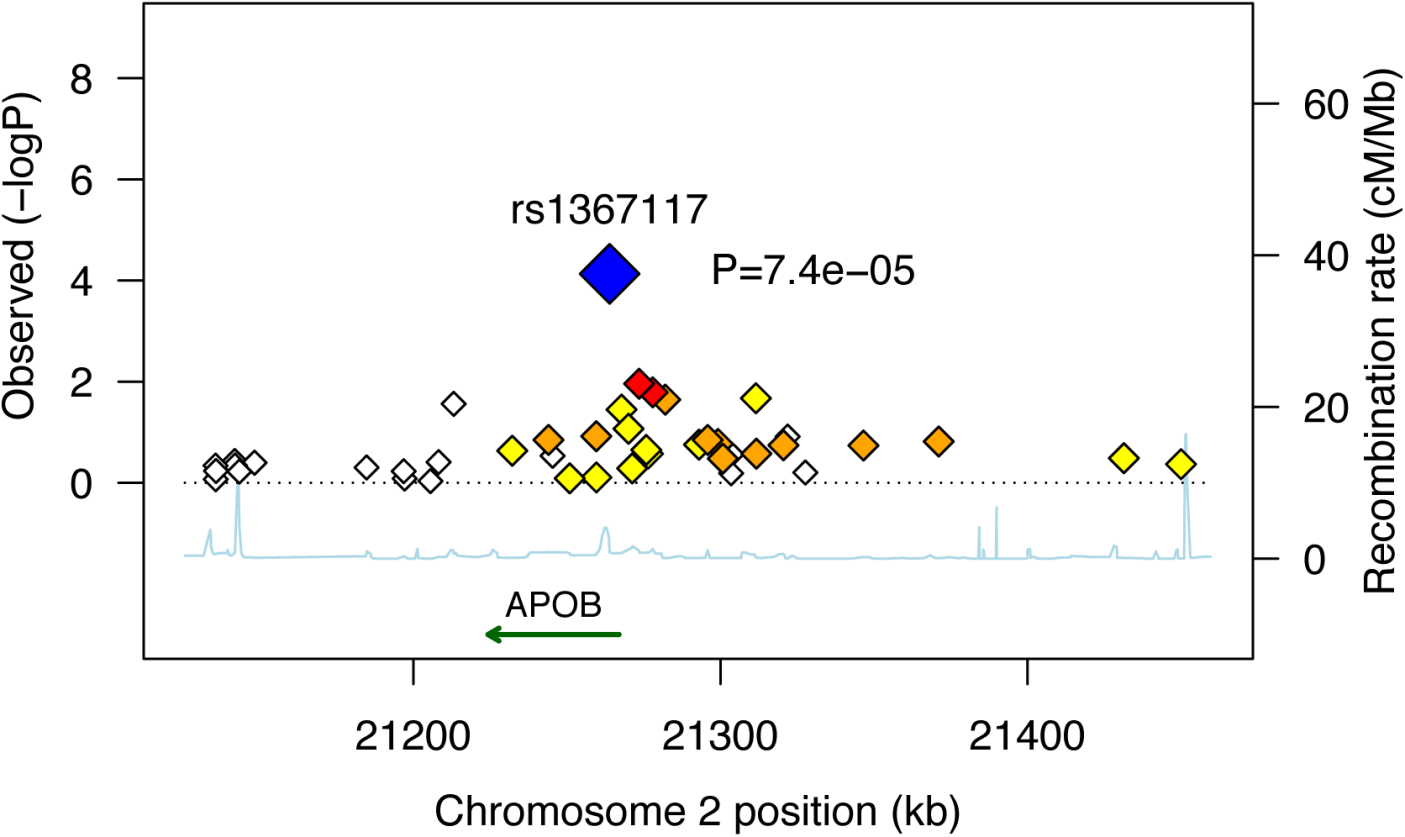
Combined* maternally-derived effects of SNPs spanning the *APOB* locus on BMI. Forty two SNPs spanning the APOB genomic locus with their corresponding meta-analysis (z-based) p-values for the maternally-derived associations (as -log10 values) are plotted as a function of chromosomal position. Estimated recombination rates are plotted to reflect the local LD structure around the associated SNP (blue) and its correlated proxies (red:R^2^≥0.8; orange:0.5≥R^2^>0.8; yellow:0.2≥R^2^>0.5; white:R^2^<0.2). Combined (Z-based) p-value for *APOB* SNP rs1367117 excluding JPS is 7.4x10^-5^ (presented in figure). Corresponding p-value including JPS is 7.8x10^-6^ (presented in Table 1). *Results used to generate the plot are based on genome-wide data available in FHS, FamHS and ERF (and not including JPS where data are not available).

In an attempt to fine-map the POE observed for *APOB* rs1367117 on adiposity, we used HapMap tagger [25] and SNAP Proxy Search function [26] to select SNPs that either tag the APOB gene region (i.e. tag SNPs using r^2^ thershold≥0.8 within the interval spanning the gene itself and 100kb on each side) or that are in moderate linkage disequilibrium (LD) with the SNP of interest (i.e. r^2^ values between 0.4–0.7). Forty two SNPs were identified and we then examined POEs of these selected SNPs on BMI in the 3 studies where genome-wide data was available (i.e. FHS, FamHS and ERF) and meta-analyzed the results (S8 and S9 Tables). To illustrate the results, we used a regional plot developed by Saxena et al. [27] (Fig 2). The plot presents the combined p-values of the maternally-derived effects on BMI, showing rather higher significance for the SNPs that are in higher LD with *APOB* rs1367117. As expected, the degree of significance of the maternal-specific associations dropped with the decrease in LD and increase in distance from the SNP of interest.

### Bioinformatics

**Fig 3:**
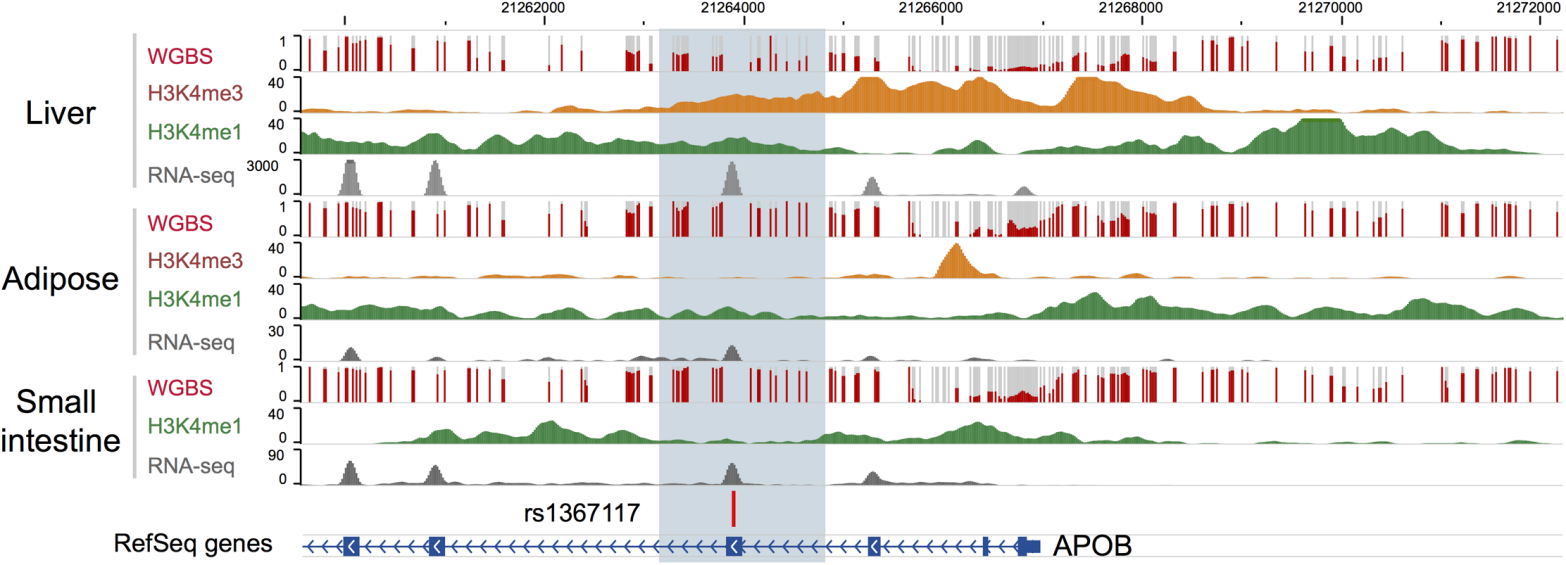
Bioinformatic annotation for the *APOB* locus. Bioinformatic annotation was undertaken for rs1367117, located within the 4th exon of the *APOB* gene, using the Epigenome Browser (http://epigenomegateway.wustl.edu/). *APOB* gene is shown in the blue track and the focal SNP with a vertical bar. Level of DNA methylation (whole genome bisulfite sequencing, or WGBS), histone marks indicative of promoters (H3K4me3) and enhancers (H3K4me1), and expression levels (RNA-seq) were plotted in three relevant tissues: liver, adipose, and small intestine.

Bioinformatic annotation was undertaken for rs1367117, located within the 4^th^ exon of apoB, using the Epigenome Browser (http://epigenomegateway.wustl.edu/; [28, 29]). The region is depicted in Fig 3. The gene is expressed most abundantly in liver, and far less in small intestine and least in adipose. In liver, there is a broad region with regulatory activity beginning upstream of the promoter and extending past the 4^th^ exon with enhancer activity around exon 4 in both liver and adipose. Therefore, this region displays a variety of regulatory functions. Notably, the DNA methylation in this region spanning exon 4 shows a 50% methylation pattern in liver, consistent with a parent-of-origin effect or allelic-specific methylation; however, the same is not true in adipose and small intestine which show nearly 100% methylation, which might indicate tissue-specific imprinting [30].

## Discussion

This study tested the hypothesis that including the parental origin of the allele will reveal parent-of-origin genetic effects on adiposity and glycemic traits among young adults. We have shown that a common coding variant in *APOB* gene has a significant maternally-derived POE on CMR traits, and adiposity in particular. Our findings for *APOB* gene provide support for the potential importance of POEs on CMR traits.

Several findings from linkage studies provide evidence for loci demonstrating POEs on CMR traits and phenotypes [13–17]. Yet despite this evidence, few genetic association studies to date have examined POEs on CMR outcomes. In a study conducted in Iceland, GWAS data were used together with genealogy and long-range phasing to examine POEs on incident Type 2 Diabetes (T2D) [18]. Importantly, the Icelandic study demonstrated that the inclusion of the parental source of offspring alleles strengthened the evidence for previously reported associations of T2D with SNPs within imprinted intervals and genes, such as KCNQ1, and revealed novel associations with T2D. Subsequently, POEs were also detected in this population of Icelanders on age at menarche and thyroid-stimulating hormone levels [19, 20], which are related to CMR traits. Other studies have shown that genetic variations in several known imprinted genes, such as *IGF2*, *INS* and *GNAS*, have been associated with CMR traits, including adult obesity (BMI) and T2D [31–36]. GWAS also have identified POEs on body composition in animal models [37, 38] and on high blood pressure [39] in humans. A novel approach for detecting POEs using genome-wide genotype data of unrelated individuals was recently introduced [40]. Using this method 6 SNPs were identified as having POE on BMI, 2 of which, near genes *SLC2A10* and *KCNK9*, were replicated in five family studies. POE was not identified for *APOB* by that study and we were not able to examine *SLC2A10* and *KCNK9* in our study as genetic data for these genes were not available in JPS.

Multiple candidate gene studies (e.g. reviewed in [41–45]) and more recently GWAS [8, 27, 46–59] identified genetic variants in *APOB* gene related to lipid and lipoprotein levels, and to coronary artery disease and myocardial infarction. Specifically, APOB rs1367117, identified here, was shown to be associated with levels of LDL-C, TC, APOB and non-HDL in GWAS [52, 54] and candidate gene studies [60–62] and recently, an epistatic interaction on BP levels was observed between *APOB* rs1367117 and *VCAM1* rs1041163 [63].

SNP rs1367117 is a nonsynonymous missense variant, located in exon 4 of *APOB* gene, causing a Thr→Ile amino acid change. A study using samples from both Finnish and Mexican subjects has shown that rs1367117 is associated with serum apoB levels, and that another variant identified in GWAS (rs7575840), which is in high LD with rs1367117 (r^2^ = 0.93 in CEU HapMap sample), is associated with expression levels of *APOB* gene in adipose tissue [61]. Obesity is strongly associated with dyslipidemia, which may account for the associated increased risk of atherosclerosis and coronary disease. Although the precise mechanism whereby obesity results in dyslipidemia has not been established, there is some evidence to support that visceral obesity is related to dysregulation of both apoB isoforms [64, 65].

Only several studies have shown that genetic variants in *APOB* gene are associated with body size and obesity in children [66] and adults [67–70] and with body growth and obesity in chicken [71]. Our results showing a highly significant additive genetic effect of *APOB* rs1367117 on lipid traits and a highly significant maternal-specific effect on adiposity traits, in fact demonstrate why genetic associations studies overlooking POE conducted to date have typically identified associations of *APOB* gene variants with lipid traits but not with adiposity.

To our knowledge, *APOB* gene has not been previously reported as demonstrating POEs on CMR or on other traits nor has it been identified as imprinted (and neither has its neighboring genes located within 500kb upstream and downstream of *APOB*, e.g. *C2orf43*, *GDF7*, *HS1BP3*, *HS1BP3-IT1*, *RHOB*, *LOC645949)*. Several explanations may account for the lack of previous support for POEs related to *APOB*. First, both imprinting and maternal genotype effects on the intrauterine environment are recognized as parent-of-origin mechanisms [10, 72], thus further work is needed to determine which of the mechanisms may explain the parent-of-origin associations identified in this work. Specifically, human placenta produces and secretes apoB-containing lipoproteins, and placental lipoprotein formation constitutes a pathway of lipid transfer from the mother to the developing fetus [73]. Whether this pathway may affect adiposity and cardio-metabolic health of adult offspring remains to be explored. Second, it is assumed that not all human imprinted genes have been identified [74–76] and gradually more genes are recognized as being imprinted [77]. Lastly, and perhaps more likely, parent-of-origin associations may be apparent in specific tissues, specific stages of development or in specific genes which are not themselves imprinted but rather regulated by imprinted genes. A recent work by Mott et al. supports the latter by showing that non-imprinted genes can generate parent-of-origin effects by interaction with imprinted loci and that the importance of the number of imprinted genes is likely secondary to their interactions [78]. It has also been suggested that imprinted genetic effects on complex traits are context dependent, and that imprinting patterns may not be consistent among traits and environments or between sexes [79]. For example, a highly significant QTL on chromosome 1, DMetS1b, was shown to be associated with variation in both serum lipid levels and obesity in mice. Interestingly, for cholesterol there was an additive effect in the full population, whereas for free-fatty acid levels high-fat fed females showed maternal expression imprinting and low-fat fed females showed paternal expression imprinting [80]. Of note, tissue and gender specificity of imprinting has been demonstrated very recently in a study in humans using allele-specific expression data [77]. Similarly, our findings in humans have demonstrated that the same *APOB* variant has an additive genotype effect on lipids, and yet a maternal POE on adiposity. In addition, context-specificity may underlie the lack of POEs of *APOB* in the ERF study, possibly due to a specific environmental factor in the Dutch population that could not be accounted for in the models. These hypotheses need to be further explored.

The major strengths of this study are the use of common genetic variation in mother-offspring pairs from a population-based cohort with quantitative CMR traits measured at age 32 and the opportunity to replicate our findings in extended pedigrees from three large family studies. Notably, the family design also minimizes confounding due to population stratification [81, 82]. Additionally, the use of candidate genes identified in GWAS takes advantage of available scientific knowledge as well as reduces the problem of multiple comparisons.

There are also several limitations to our study. First, genetic data on fathers were unavailable in JPS resulting in the use of probability-based imputation of the parental source of minor alleles in heterozygous mother-offspring pairs. However, as we have shown in sensitivity analyses, this approach is likely conservative and therefore preferable compared to excluding heterozygous mother-offspring pairs. Furthermore, replication in other studies where genetic data in extended families were used minimizes this source of bias. Second, the *APOB* variant indentified here has not been previously reported in large GWAS consortia as related with adiposity. Although this is likely the result of GWAS typically ignoring the parental source of alleles due to lack of family data, there is a possibility that our POE results reflect false-positive findings. To address this, we have conducted simulations applying two different approaches, one for a mother-offspring design (using JPS) and the other for an extended pedigrees design (using FHS). Based on these simulations, maternal permutation p-values were similar to our observed p-values providing further support for the significance of the reported maternal *APOB* effect. Lastly, environmental exposures around the time of birth may contribute to the context-specificity of POEs. These data were unavailable for most of the participating studies and therefore their effects could not be assessed in this work.

In summary, we have demonstrated that taking into account the parental origin of offspring alleles compared to examining the offspring genotype as a whole may enhance our understanding of genetic associations with CMR traits. Our results highlight the potential contribution of POEs to uncovering complex relations between genetic variants and common traits, and motivate further research in this area, including assessment of the impact of POEs on common traits and investigation of the mechanisms underlying these associations.

## Methods

The Jerusalem Perinatal Study (JPS) population-based cohort includes a sub-cohort of all 17,003 births to residents of Jerusalem, between years 1974 and 1976 [83–85]. Data consist of demographic and socioeconomic information, medical conditions of the mother during current and previous pregnancies, and offspring birth weight, abstracted either from birth certificates or maternity ward logbooks. Additional information on lifestyle and maternal medical conditions, including gestational age, mother's smoking status, height and pre-pregnancy weight, end of pregnancy weight and gynecological history, was collected by interviews of mothers on the first or second day postpartum. Data collection methods have been described in detail previously [83–85].

The Jerusalem Perinatal Family Follow-Up Study includes a sample of 1,250 mother-offspring pairs (average offspring age 32, range: 30–35) from the original 1974–1976 birth cohort, who were interviewed and examined between 2007 and 2009. Information on sampling and data collection in the JPS Family Follow-Up study was previously described [86]. Briefly, the sampling frame included singleton term births without congenital malformations, and a stratified sample defined by maternal pre-pregnancy body mass index (BMI) and birth weight was obtained, where both low and high birth weight as well as over-weight and obese mothers were over-sampled. Standard procedures and training protocols were used to measure standing height, body weight, waist circumference and blood pressure. Blood specimens collected after fasting for at least 8 hours were taken using standard procedures, and biochemical measurements of insulin, glucose, cholesterol, HDL-C and triglycerides were assayed in plasma. Inter assay coefficients of variation (CV%) were less than 2% for glucose and less than 2.5% for cholesterol, HDL-C and triglycerides.

Individuals who reported taking medication to treat diabetes (n = 13), lipid-lowering medication (n = 13) or BP-lowering medication (n = 11), were excluded from the corresponding analyses.

### DNA extraction and genotyping

Blood was collected in tubes containing EDTA. DNA was extracted from white blood cells using standard salting-out extraction procedures. Common tag SNPs from candidate genes in molecular pathways potentially related to both birth weight and CMR phenotypes were previously genotyped in mother-offspring pairs using Illumina 1536 SNPs panel (BeadArray technology with a GoldenGate custom panel, Illumina, San Diego, CA, USA [87, 88]). Of the 1536 SNPs genotyped, 1384 (90.1%) provided genotype clusters with call rates>95%. Additional quality control measures for genotypic data included: 1) testing Hardy-Weinberg Equilibrium; SNPs with chi-square p-value < 0.05/1384 were excluded; and 2) observing Mendelian inheritance inconsistency between mother’s and offspring genotypes; SNPs with more than 0.5% inconsistency were excluded. As a result, an additional 10 SNPs were removed and a total of 1374 SNPs, representing 180 genes, were available.

### SNP selection

To identify POEs on adiposity and glycemic traits we selected SNPs within genes where previous GWAS in Caucasian populations had revealed variants associated with BMI, glucose and insulin levels, lipids and lipoproteins levels, obesity and type 2 diabetes. This is similar to the approach applied in the Icelandic study in the sense that published GWAS findings were used for targeting variants of interest for POE analysis [18]. Specifically, using the US National Institutes of Health Office of Population Studies catalogue of published genome-wide association studies (www.genome.gov/gwastudies—accessed July 1, 2012) [89], out of the 180 genes genotyped previously using the Illumina custom panel in JPS mother-offspring pairs, we identified 18 genes for which significant associations (p-value<5X10^-8^) with CMR traits were reported in GWAS (Table 4 and S1 and S2 Tables). In these 18 genes 182 tag SNPs were available in JPS (Table 4 and S3 Table).

**Table 4:** Genes selected for examination of parent-of-origin effects in JPS.

#	Gene	Gene ID	chr	Location (build GRCh37/hg19)	# genotyped SNPs
1	*APOB*	338	2	chr2:21224301–21266945	10
2	*CDKN2A*	1029	9	chr9:21967751–21994490	5
3	*CDKN2B*	1030	9	chr9:22,008,716–22,008,952	4
4	*FTO*	79068	16	chr16:53737875–54148379	2
5	*GCK*	2645	7	chr7:44183870–44229022	5
6	*HNF4A*	3172	20	chr20:42,984,441–43,061,485	9
7	*IGF1*	3479	12	chr12:102,789,645–102,874,378	10
8	*IRS1*	3667	2	chr2:227596033–227663506	1
9	*LPL*	4023	8	chr8:19796582–19824770	11
10	*MC4R*	4160	18	chr18:58038564–58040001	1
11	*MTHFR*	4524	1	chr1:11,845,787–11,866,160	9
12	*POMC*	5443	2	chr2:25383722–25391559	4
13	*PPARG*	5468	3	chr3:12,328,984–12,475,855	13
14	*PSRC1*	84722	1	chr1:109,822,176–109,825,808	3
15	*TCF1 (HNF1A)*	6927	12	chr12:121,416,549–121,440,314	8
16	*TCF2*	6928	17	chr17:36,046,434–36,105,096	22
17	*TCF7L2*	6934	10	chr10:114,710,009–114,927,436	42
18	*TUB*	7275	11	chr11:8,040,791–8,127,654	23

### Cardio-metabolic outcomes

The following adiposity and glycemic traits were measured in offspring at age 32: body mass index (BMI, calculated by dividing weight (kg) by squared height (m^2^)); waist circumference (WC, mean of two consecutive measurements at the midpoint between the lower ribs and iliac crest in the midaxillary line (cm)); fasting glucose (mg/dL) and fasting insulin (mean of two repeated measures (mU/mL), natural log-transformed).

In a follow-up analysis limited to replicated findings, we additionally examined lipid and blood pressure traits: low-density lipoprotein cholesterol (LDL-C (mmol/L)); high-density lipoprotein cholesterol (HDL-C (mmol/L)), triglycerides (TG (mmol/L), natural log-transformed), systolic and diastolic blood pressure (SBP and DBP, mean of three consecutive measures (mmHg)).

All cardio-metabolic outcomes were treated as continuous variables (distributions presented in S4 Table).

### Statistical analyses

Analyses of JPS data were carried out using Stata 12.0 (StataCorp, College Station TX).

**Fig 4:**
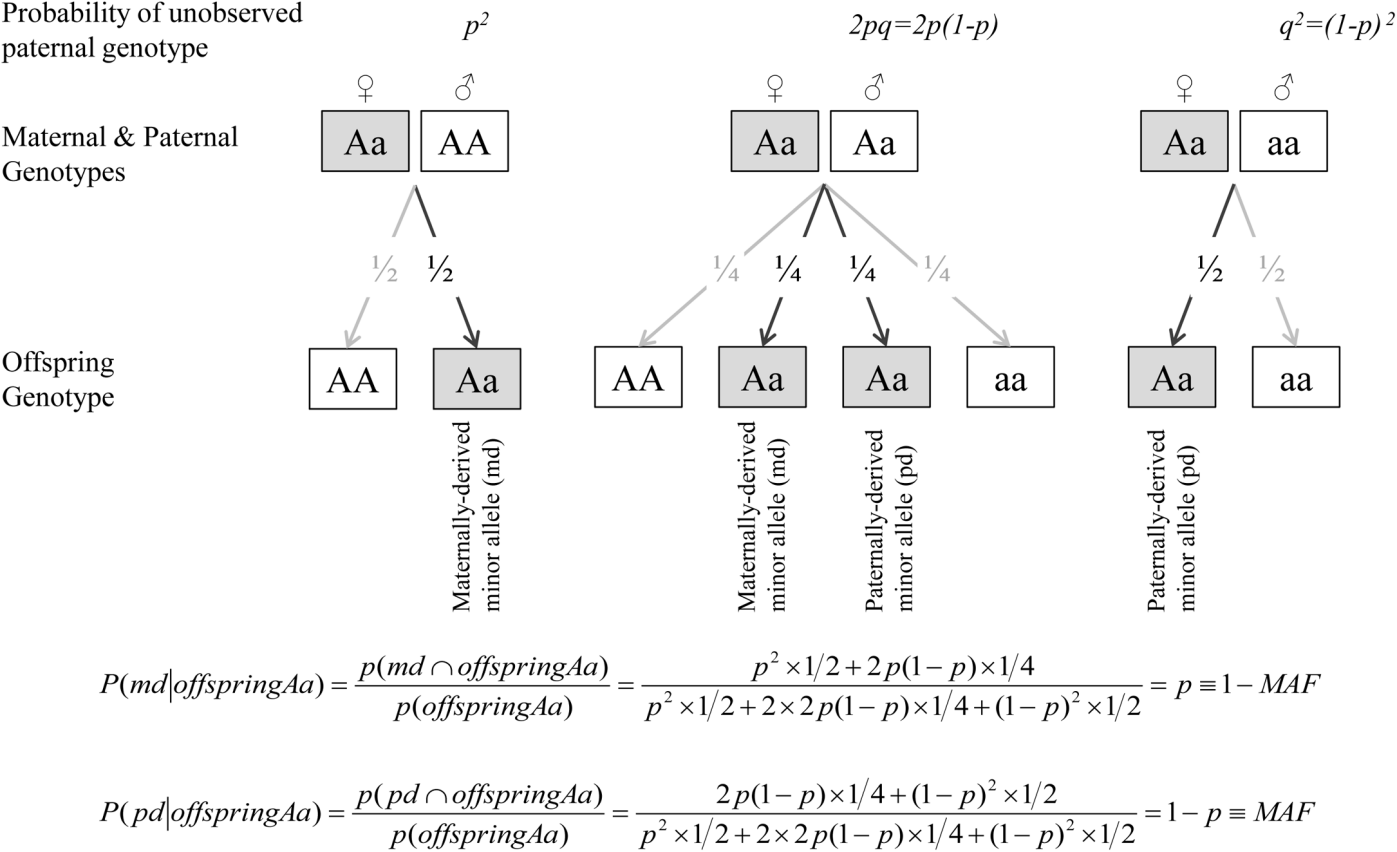
Estimated dosage of maternally- and paternally-derived minor allele indicators for heterozygous (Aa) mothers-offspring pairs.

To assess the separate contribution of the maternally- and paternally-inherited variants to offspring CMR phenotypes, we first determined the parental origin of minor alleles, based on mother-offspring pairs, since genetic data on fathers were not available in JPS. For every given SNP we constructed two variables; maternally-derived minor allele (M-D) and paternally-derived minor allele (P-D) indicators. These indicators count the number of minor alleles inherited from mother and/or father; each count is a zero or one (Table 4). When offspring and mother were both heterozygous at a given SNP, the source of the minor allele was ambiguous. But under random mating and transmission equilibrium, the probability that the minor allele was derived from the mother is 1-MAF and from the father is MAF, where MAF = minor allele frequency. Therefore, similarly to use of estimated dosage for uncertain genotypes, for heterozygous mothers-offspring pairs, we used the estimated dosage of M-D and P-D indicators, i.e. 1-MAF and MAF, respectively (Table 5 and Fig 4). The percentage of heterozygous mother-offspring for each of the 182 selected SNPs ranged between 5% and 27% (S3 Table).

**Table 5:** Indicators of parental origin of the minor allele in JPS.

mother genotype	offspring genotype	M-D indicator	P-D indicator
AA	AA	0	0
	Aa	0	1
Aa	AA	0	0
	Aa	1-MAF	MAF
	aa	1	1
aa	Aa	1	0
	aa	1	1

When offspring and mother are both heterozygous at a given SNP, the source of the minor allele is ambiguous. For any given SNP with allele frequencies of p and q for the common (A) and minor (a) alleles respectively, the expected paternal genotype frequencies assuming Hardy-Weinberg Equilibrium are *f*(AA) = *p* ^2^, *f*(Aa) = 2*pq and f*(aa) = *q* ^2^. Under random mating and transmission equilibrium, the probability that the minor allele was derived from the mother is 1-MAF and from the father is MAF, where MAF = minor allele frequency. Therefore, for heterozygous mothers-offspring pairs, we used the estimated dosage of M-D and P-D indicators, i.e. 1-MAF and MAF.

We used linear regression to examine each SNP-trait association. We first assessed the associations of offspring genotype with trait, using an additive genetic model. Then the association of the parental origin of the minor allele on the trait was assessed by including the M-D and P-D indicators together in a single model.

The following mean models were used:

$$E[Y]=\beta_{0}+\beta_{G}G_{O}+\gamma^{T}Z$$ (1)

$$E[Y]=\beta_{0}+\beta_{MD}G_{MD}+\beta_{PD}G_{PD}+\gamma^{T}Z$$ (2)

Where Y denotes trait, *G*_*O*_ = offspring genotype, *G*_*MD*_ = indicator of maternally-derived minor allele, *G*_*PD*_ = indicator of paternally-derived allele, and Z = other covariates.

Existence of POE in model 2 was assessed via a test of the following null hypotheses:

(1) *β*_*MD*_ = 0; (2) *β*_*PD*_ = 0; (3) *β*_*MD*_ = *β*_*PD*_. The significance threshold for rejection of the null hypotheses was set at a within-gene corrected Bonferroni p-value<0.05 for the separate effects of the maternally- or paternally derived indicators (i.e. hypotheses 1 or 2) and nominal p-values (i.e. p-values<0.05) for the F-test examining the differences between these effects (i.e. hypothesis 3). Findings that met these criteria for any one of the examined outcomes were moved forward for replication and meta-analysis.

All models were adjusted for offspring sex and ethnicity. Following an approach suggested by Thomas and Witte to correct for population stratification in mixed-ethnicity families [90], ethnicity of offspring was classified based on country of origin of all four grandparents, using nine major ethnicity strata (Israel, Morocco, Other North Africa, Iran, Iraq, Kurdistan, Yemen, Other Asia and the Balkans and Ashkenazi). Rather than allocating offspring to a single ethnicity, we constructed a covariate for each stratum giving the proportion of grandparents derived from each of the nine ethnic groups (ranging from 0 to 1, reflecting none or all four grandparents originating from the specific ethnic group, respectively) and then included these covariates as adjustment variables in a multiple regression, excluding one stratum (Ashkenazi) to eliminate complete multicollinearity.

To account for the stratified sampling, we used Stata’s ‘pweight’ option to weight estimates by individuals’ inverse probability of being sampled [91].

### Replication and meta-analysis

Findings exceeding the aforementioned threshold of significance in JPS were followed up in three additional family studies with extended pedigrees: 1) Framingham Heart Study (FHS) (max N = 2225 probands); 2) Family Heart Study (FamHS) (max N = 773 probands); and 3) Erasmus Rucphen Family (ERF) study (max N = 631 probands). Probands were restricted to individuals of European decent, aged≤50, with available genetic and CMR data and genetic data in at least one parent. Probands on medication to treat diabetes, lipid-lowering medication or BP-lowering medication were excluded from the corresponding analyses. Detailed description of each of the studies, including study sample, genotyping techniques and CMR trait measurements and distributions, is provided in S1 Text and S4 Table. Distributions of CMR traits were comparable between the studies, with the exception of fasting insulin in FHS. Yet substantial differences in fasting insulin distributions across studies are commonly observed [92, 93]. We used the quantitative transmission disequilibrium test (QTDT) software [94] to test for the association of maternally and paternally inherited minor alleles using information from the extended families within each study, adjusting for sex and age, using a model similar to the one used in JPS. Specifically, qtdt -at -ot was used to test for POE (maternal effect = paternal effect), and qtdt -at -om and qtdt -at -op were used to test maternal and paternal effects, respectively (modified to adjust for other parent contribution). QTDT software uses a linear mixed effect model to account for familial correlations. We did not use the QTDT statistic that is robust to population stratification because, based on available genome-wide data, population stratification did not affect the traits evaluated in most studies. In limited instances where there was some suggestive evidence for the presence of population stratification, we corrected for it using principal components analysis within the relevant study [95].

Additionally, z-based meta-analysis combining results from all 4 studies (i.e. JPS, FHS, FamHS and ERF) was conducted, as a joint analysis approach was shown to be more efficient than a two-stage approach for genetic association studies [96]. A Bonferroni correction was applied and SNPs were declared statistically significant if their p-values were below 0.05 divided by the total number of SNPs initially tested (N = 182), i.e. p-value<2.75x10^-4^. We also applied an inverse variance weighted meta-analysis approach to obtain a pooled estimate of effect sizes (betas) across studies. Since the QTDT approach does not provide estimates of standard errors (SEs), SEs were estimated by converting p-values into a z-statistic and setting: SE = beta/z.

### Ethics statement

The JPS study was approved by the Institutional Review Board of the Hadassah-Hebrew University Medical Center (approval #10–01.04.05) and the University of Washington Human Subject Review Committee (approval #31032). FHS was approved by Boston University Medical Center Institutional Review Board (approvals #H–32132, #H–26671 and #H–28859), FamHS was approved by the Institutional Review Board of the Washington University School of Medicine in St. Louis (approval #201403014) and ERF study was approved by the Medical Ethics Committee of Erasmus MC, Rotterdam (approval #MEC 213.575/2002/114). All participants provided written informed consent for genetic studies.

## Supporting Information

S1 Text 1) Description of replication studies—Short description of FHS, FamHS, ERF, including available genetic data and CMR measurements. 2) Permutation testing—Description of methods and results of permutations conducted in JPS and FHS. 3) Acknowledgments by study. (DOC)

S1 Table Genes identified in GWAS of CMR traits and available in JPS. (XLSX)

S2 Table Search results of the GWAS catalog (accessed July 1, 2012) for the selected JPS genes. (XLSX)

S3 Table List of SNPs analyzed in discovery phase in JPS. (XLSX)

S4 Table Distribution of CMR traits by study. (XLSX)

S5 Table Discovery phase results (JPS). (XLSX)

S6 Table Replication and meta-analysis results for 30 SNPs identified in JPS. (XLSX)

S7 Table Parent-of-origin effects of APOB SNP rs1367117 on CMR traits, with and without further adjustment for BMI. (XLSX)

S8 Table Information on 42 SNPs in APOB region in FHS FamHS and ERF. (XLSX)

S9 Table Meta-analysis results of parent-of-origin effects of 42 SNPs spanning the APOB locus in FHS, FamHS and ERF. (XLSX)
