# Supplementary material for: Parent-of-Origin Effects of the APOB Gene on Adiposity in Young Adults
Source: PLoS Genet. 2015 Oct 9;11(10):e1005573. doi: 10.1371/journal.pgen.1005573 (PMC4599806; doi:10.1371/journal.pgen.1005573)
Supplement: S1 Text — (DOC) [file pgen.1005573.s001.doc]

**1) Description of replication studies**

**Framingham Heart Study (FHS)**

Cohort description

In 1948, the FHS was initiated and the Original cohort was formed with 5,209 participants from Framingham, Massachusetts, US. Participants have attended exams roughly every two years to investigate cardiovascular disease and related risk factors. In 1971, a second cohort, the Offspring cohort, was recruited and included 5,124 children of the Original cohort and the children’s spouses. Offspring participants have attended exams approximately every four years. The Third Generation cohort was recruited in 2002 and includes 4,095 children of the Offspring cohort who have completed two exams. Included in the current study are 559 Offspring participants who were 50 years or younger at exam 5, and 3294 Generation 3 participants age 50 or younger at study entry, who also had at least one parent with genotypes available.

Genotyping

Genotypes were from the CARe and SHARe project (dbGAP weblink: http://www.ncbi.nlm.nih.gov/projects/gap/cgi-bin/study.cgi?study_id=phs000007.v25.p9). In brief, genotypes from Affymetrix 500K SNP arrays supplemented with the MIPS 50K array were available. A subset of 378,163 SNPs passing stringent filters including a minor allele frequency ≥ 0.01, SNP call rate ≥ 0.97, HWE p-value ≥ 1e-6, differential missingness p-value ≥ 1e-9 and < 100 Mendelian errors were used for imputation based on the haplotypes of the HapMap CEU trios using the MACH software.

Trait measurements

Fasting glucose and insulin were measured after a fast of 8 or more hours in plasma using Hexokinase reagent kit (a-gent glucose test, Abbott, South Pasadena, California) for glucose and DPC Coat-A-Count RIA (total immunoreactive insulin) for insulin. Waist circumference was measured at the level of the umbilicus to the next lower ¼ inch. BMI was calculated by taking the weight (measured to nearest pound and converted to kilograms) over the height (measured to next lower ¼ inch, converted to meters) squared. Blood lipids (total cholesterol, HDL-C, and TG) were measured by standard enzymatic methods. LDL-C was calculated using the Friedewald formula, setting to missing participants with TG > 400 mg/dL. At each clinic visit, the examining physician measured the systolic and diastolic BP in the left arm using a mercury column sphygmomanometer. BP was measured twice by a physician. Systolic and diastolic pressures were determined by the first and fifth Korotkoff sounds, respectively, and the two BP measurements were averaged to derive the systolic and diastolic pressures for that examination. In the Generation 3 participants, all trait values were taken from the first exam. In the Offspring participants, fasting glucose, fasting insulin, waist and BMI were measured at the 5^th^ exam, while lipid levels and blood pressure were measured at the first exam.

**Family Heart Study (FamHS)**

Cohort description

The Family Heart Study began in 1992 with the ascertainment of 1,200 families (6,000 individuals), half randomly sampled, and half selected because of an excess of coronary heart disease (CHD) or risk factor abnormalities as compared with age- and sex-specific population rates . The families were sampled from four population-based parent studies: the Framingham Heart, the Utah Family Tree, and two ARIC centers (Minneapolis, University of Minnesota, and Forsyth County, NC, University of North Carolina). The proband, his or her spouse, children, brothers, sisters, and parents were recruited, thus producing three-generation pedigrees. These subjects participated in a clinic visit, after providing appropriate informed consent. A broad range of phenotypes was assessed including anthropometry, and parameters of glucose metabolism, lipids, and blood pressure. Only subjects of European descent were used in this study, including a total of 2,756 subjects in 510 extended families.

Genotyping

Genotypes assessed with chip arrays were available for 971 subjects on the Illumina HumMap 550K, 1,674 subjects on the Illumina Human 610-Quadv1, and 1,490 subjects on the Human 1M-Duov3 Illumina chip. Genotyping quality control was performed before imputation, and typed SNPs were included if call rate >0.98; minor allele frequency (MAF) >0.01; no deviation from Hardy-Weinberg equilibrium (HWE p > 1x10^-06^); and provided the SNP was present in the 1000 Genomes Project Phase I (1000G) reference panel. Additionally, an assessment of Mendelian errors and the verification of reported pedigree relationships using GRR were conducted . Imputation was performed by genotyping platform using 1000G reference panel (including all races—version 2010-11 data freeze, 2012-03-04 haplotypes). Pre-phasing was carried out prior to imputation and SNPs with imputation quality score (r2) value > 0.5 were included in the analyses to assure high quality imputation. Because dosages cannot be used in POE analyses, the imputed dosages were transformed to the best guess genotype using stringent criteria (dosage >1.9 was homozygous coded allele, dosage between 0.9 and 1.1 was heterozygous, and dosage <0.1 was homozygous for the alternative allele, otherwise the SNP call was set to missing).

Trait measurements

BMI was calculated as weight (in kg) divided by the square of height (in meters). Waist girth was measured at the level of the umbilicus and was recorded to the nearest centimeter, rounding down at the point of relaxed end exhalation.

Glucose was measured by a thin film adaptation of a glucose oxidase enzymatic, spectrophotometric procedure using the Vitros analyzer (Ortho Clinical Diagnostics, Rochester, NY). Insulin measurement was performed by the coated-tube radioimmunoassay method distributed by Diagnostic Products Corp. (Los Angeles, CA). Following the analysis plan, glucose and insulin measures were included if participants fasted for at least 8 hours. Additionally, participants being treated for diabetes and/or who had fasting glucose>126 mg/dl were excluded.

Lipids were measured by standard enzymatic methods. Following the analysis plan, participants with TG>400 and participants on lipid lowering medications were excluded.

Blood pressure was measured with the patients in a sitting position after a resting period of at least 5 minutes. Three measurements with a random-zero sphygmomanometer were taken. The mean of the systolic and diastolic blood pressure measurements was used. Participants on blood pressure lowering medications were excluded.

**Erasmus Rucphen Family (ERF)**

Cohort description

ERF is embedded in the Genetic Research in Isolated Population (GRIP) program. GRIP is a Dutch isolated population located in the southwest of the Netherlands. The Genealogy of GRIP is available from its founding (1600-1650). For ERF, we selected 22 related (first and second degree, non-consanguineous) couples living in GRIP between 1850-1900, and having at least six children. All living descendants of those couples were invited to participate in ERF. The 3000 participants aged between 18-20 years constitute a unique single family spanning 23 generations consisting of over 23,000 individuals. All participants gave written Informed consents and the study was approved by the Medical Ethics Committee of Erasmus MC, Rotterdam.

Genotyping

3,685 individuals from ERF and GRIP were genotyped on various genome-wide genotyping arrays including Illumina 300K, Illumina 370k, Illumina 610K and Affymetrix 250K. Quality control was performed on each set separately. All variants with a call rate of less than 95% or were out of Hardy-Weinberg equilibrium at a p-value < 10^-06^ or had excess heterozygosity at fdr < 1% were excluded. Individuals with a call rate less than 95%, duplicates and ethnic outliers (with PCA with HapMap) were excluded. All datasets were merged after QC and genotypes were imputed from the 1000 genomes Phase I V3 using minimac. Pre-phasing was carried out prior to imputation and monomorphic imputed variants were excluded. Further 1,527 from ERF were additionally genotyped on the Illumina Infinium HumanExome BeadChip, version 1.1, which contains over 240,000 exonic variants selected from multiple sources together spanning 12,000 samples from multiple ethnicities. Calling was performed with GenomeStudio and the ZCall variant calling tool (Broad Institute) . We removed subjects with a call rate < 0.95, IBS > 0.99 and heterozygote ratio > 0.60, and SNPs that were monomorphic in our sample or had a call rate < 0.95. After QC we retrieved 70,138 polymorphic SNVs, and 1,512 subjects to be included in the analysis. Variants with Mendelian errors were set to missing in the parents and their children.

Exome-Sequencing

1,336 subjects from the ERF study were sequenced “in-house” at the Center for Biomics of the Cell Biology department of the Erasmus MC, The Netherlands. These subjects were selected for having good quality phenotype information on a wide range of topics, and therefore random with regards to cognitive test scores. The sequencing was performed using the Agilent version V4 capture kit on an Illumina Hiseq2000 sequencer using the TruSeq Version 3 protocol. The sequence reads were aligned to the human genome build 19 (hg19) using BWA and the NARWHAL pipeline. Subsequently, the aligned reads were processed further using the IndelRealigner, MarkDuplicates and TableRecalibration tools from the Genome Analysis Toolkit (GATK) and Picard (http://picard.sourceforge.net) to remove systematic biases and to recalibrate the PHRED quality scores in the alignments. For each sample, at least 4 Gigabases of sequence was aligned to the genome. Annotations were performed using the dbNSFP (database of human non-synonymous SNPs and their functional predictions) and Seattle databases available at: <http://snp.gs.washington.edu/SeattleSeqAnnotation131/>. These databases gave functional prediction results from five different programs including polyPhen2, MutationTaster, SIFT, MutationAssessor and LRT, apart from gene and variant annotations, as well as conservation scores (Grantham score, PhyloP). In total 1,415,9934 Single Nucleotide Variants (SNVs) were called. After removing the low quality variants (QUAL < 150), variants with low (< 95%) call rate and out of Hardy-Weinberg equilibrium (p-value < 10^-06^); and excluding swapped samples and those with low call rate (< 88%) we retrieved 540,633 high-quality SNVs in 1,301 individuals. Further, variants with Mendelian errors were set to missing in the parents and children.

Trait measurements

All descendants were invited to visit the regional clinical research centre where they were examined and a fasting blood sample was drawn. Blood from participants was obtained in a fasted state. Total plasma insulin measurements were analyzed with an INS-IRMA kit (BioSource). Height and weight were measured with the participant dressed in light underclothing. BMI was calculated from these data. Waist circumference was measured on uncovered skin using a tape measure with the participant in the upright position halfway between the rib cage and the pelvic bone. Participants were asked to fast for at least 8 hr prior to blood withdrawal. Blood samples were then sent to the local laboratory for standard plasma glucose and serum lipid measurements, which were made using a Synchron LX 20 Systems analyser (Beckman Coulter, Fullerton, CA, USA). Blood pressure in the right arm was measured while sitting, using an automated device (OMRON HEM-711; OMRON Healthcare, Vernon Hills, IL, USA) after at least 5 min of rest. The average of two measurements taken 5 min apart was used for analysis. Following the analysis plan, probands on medication to treat diabetes, lipid-lowering medication or blood pressure-lowering medication were excluded from the corresponding analyses.

**2) Permutation Testing**

To address the possibility that the reported *APOB* POE results reflect false-positive findings, we have conducted simulations applying two different approaches, one for a mother-offspring design (using JPS) and the other for an extended pedigrees design (using FHS).

In the JPS, we randomly permuted offspring adiposity traits (i.e. BMI and waist circumference) 10,000 times within each gender stratum to generate an empirical distribution of p-values for the maternal and paternal effects, under the null hypothesis of no POE, using linear regression. We then determined the probability of our POE findings by dividing the number of empirical p-values that are smaller than our observed p-values by 10,000.

In FHS, due to the extended pedigree structure, we implemented an approach described by Iturria et al . Under this approach, adiposity traits (i.e. BMI and waist circumference) were simulated from a multivariate normal model with a 50% heritability, similar to what has been reported in the literature for waist circumference and BMI, under the constraint of no POE effect. Then the ranking of the simulated traits were paired with the observed traits to perform a permutation of observed values in a way that maintains much of their familial correlation, and hence the heritability of the trait, which is desirable since the goal is to assess the statistical significance of the observed POE finding in the presence of background genetic variation. P-values for the maternal and paternal effects were recomputed 10,000 times using QTDT, and P-values for the original POE findings were calculated as the proportion of permutations based p-values that were smaller than the original p-values.

Results for these permutations in JPS and FHS are presented in the table below. Generally, in both JPS and FHS observed and permuted p-values for the maternal and paternal effects of the *APOB* SNP rs1367117 on BMI and waist were very similar, pointing to a significant maternal and not paternal effect of this SNP on adiposity.

|  | Maternal effect | Paternal effect |  |  | Maternal effect | Paternal effect |
| --- | --- | --- | --- | --- | --- | --- |
| **BMI** | | |  | **Waist Circumference** | | |
| **JPS** |  |  |  | **JPS** |  |  |
| Observed p-value | 0.0065 | 0.9927 |  | Observed p-value | 0.0027 | 0.6049 |
| Permutation p-value | 0.0079 | 0.9939 |  | Permutation p-value | 0.0048 | 0.6165 |
| **FHS** |  |  |  | **FHS** |  |  |
| Observed p-value* | 0.0014 | 0.3248 |  | Observed p-value* | 0.0006 | 0.6316 |
| Permutation p-value | 0.0059 | 0.3854 |  | Permutation p-value | 0.0029 | 0.6833 |

**3) Acknowledgments by study**

Funding for JPS was provided by NIH research grant R01HL088884 (Dr. Siscovick), the Israeli Science Foundation grants No. 1252/07, 552/12 (Dr. Friedlander) and partly by the NUS-HUJ CREATE Programme of the National Research Foundation, Singapore (Project Number 370062002, Dr. Friedlander).

Funding for FHS was provided by National Institute for Diabetes and Digestive and Kidney Diseases (NIDDK) 2R01DK078616, K24 DK080140 (Dr. Meigs), and the National Heart, Lung and Blood Institute's Framingham Heart Study (Contract Nos. N01-HC-25195, N02-HL-6-4278). A portion of this research was conducted using the Linux Clusters for Genetic Analysis (LinGA) computing resources at Boston University Medical Campus.

The work in FamHS was partly supported by an NIDDK grant R01DK8925601 (Dr. Borecki).

The ERF Study was supported by the joint grant from the Netherlands Organization for Scientific Research (NWO, 91203014), the Center of Medical Systems Biology (CMSB), Hersenstichting Nederland, Internationale Stichting Alzheimer Onderzoek (ISAO), Alzheimer Association project number 04516, Hersenstichting Nederland project number 12F04(2).76, and the Interuniversity Attraction Poles (IUAP) program.The ERF study as a part of EUROSPAN (European Special Populations Research Network) was supported by European Commission FP6 STRP grant number 018947 (LSHG-CT-2006-01947) and also received funding from the European Community's Seventh Framework Programme (FP7/2007-2013)/grant agreement HEALTH-F4-2007-201413 by the European Commission under the programme "Quality of Life and Management of the Living Resources" of 5th Framework Programme (no. QLG2-CT-2002-01254). High-throughput analysis of the ERF data was supported by joint grant from Netherlands Organization for Scientific Research and the Russian Foundation for Basic Research (NWO-RFBR 047.017.043). Exome sequencing analysis in ERF was supported by the ZonMw grant (project 91111025). Exome-chip genotyping was supported by BBMRI-NL. We are grateful to all study participants and their relatives, general practitioners and neurologists for their contributions and to P. Veraart for her help in genealogy, J. Vergeer for the supervision of the laboratory work and P. Snijders for his help in data collection.

**References:**

1. Higgins, M., et al., *NHLBI Family Heart Study: objectives and design.* Am J Epidemiol, 1996. **143**(12): p. 1219-28.

2. Abecasis, G.R., et al., *GRR: graphical representation of relationship errors.* Bioinformatics, 2001. **17**(8): p. 742-3.

3. Goldstein, J.I., et al., *zCall: a rare variant caller for array-based genotyping: genetics and population analysis.* Bioinformatics, 2012. **28**(19): p. 2543-5.

4. Iturria, S.J., et al., *An empirical test of the significance of an observed quantitative trait locus effect that preserves additive genetic variation.* Genet Epidemiol, 1999. **17 Suppl 1**: p. S169-73.
